# A Lilium Tigrinum Case

**Published:** 1889-06

**Authors:** E. W. Berridge

**Affiliations:** London


### A LILIUM TIGRINUM CASE.

E. W. Berridge, M. D., London.

June 23d, 1887.—A lady aged fifty or more consulted me for the following symptoms: Pain in apex of heart, as if grasped by the hand, preceded and accompanied by a cold feeling, extending from the apex of heart to under left scapula; a spot at apex of heart, size of finger-tip, is tender to pressure. This heart trouble has been very bad for a week, and for a longer time has had it less severely; she had it also years ago. The pain is excited by worry; it is worse on lying on right side, *better by lying on left side and when busy at work.* Constant hacking cough; every morning attack of asthma during breakfast, and lasting two hours, caused by feeling of hard pressure at lower end of sternum; she has had this asthma at intervals of six or seven weeks, daily for the last two weeks. Constant desire to draw long breath and sigh (the most recent symptom). When walking, and less often when sitting, the right leg from hip to foot turns in; with this symptom, she feels a weakness all down right side, right arm, and right leg, and at times slight tingling and burning in right upper arm and all down right leg; she has had this symptom for two weeks, and years ago used to have it. For a few weeks, dryness of vagina, the parts seem to rasp together when walking, with occasional sharp, stinging pain. I gave *Lilium tigrinum*$^{cm}$ (Fincke) one dose, and a few more doses to be taken if the symptoms returned in the same form and persisted.

I did not see her for about a year, when she told me the remedy had cured all the symptoms. She could not remember if she had to repeat the dose; but was certain that not more than two or three doses, at the most, were required.

The following symptoms were also cured in the same patient:

July 15th, 1885.—*Alumina*$^{cm}$ (F. C.) relieved an intense dragging, burning pain in back of eyes, with intense photophobia; also a blaze of light before the closed eyes; attacks of pain worse at three A. M. The pain improved before the photophobia.

Nov. 2d, 1885.—Attacks of pain beginning in left side, sometimes going round to left side of back, on waking in morning; and when she begins to move, the flesh there feels torn from the bones, slightly relieved by eructations, though the eructations cause spasms across spleen and round stomach. *Thuja*$^{cm}$ (F. C.) cured "like a shot."

Feb. 25th, 1886.—*Tellurium*$^{31}$ in water, two or three times daily for four days, removed a feeling as if the lashes of lower lids were turned in.

